# The Impact of Photobleaching on Microarray Analysis

**DOI:** 10.3390/biology4030556

**Published:** 2015-09-11

**Authors:** Marcel von der Haar, John-Alexander Preuß, Kathrin von der Haar, Patrick Lindner, Thomas Scheper, Frank Stahl

**Affiliations:** Institute of Technical Chemistry, Leibniz University Hanover, Callinstr. 5, 30167 Hanover, Germany; E-Mails: johnalexanderpreuss@googlemail.com (J.-A.P.); vonderhaar@iftc.uni-hannover.de (K.H.); lindner@iftc.uni-hannover.de (P.L.); scheper@iftc.uni-hannover.de (T.S.); stahl@iftc.uni-hannover.de (F.S.)

**Keywords:** microarray, DNA, photobleaching, fluorophore, cyanine dye, bioinformatics, bioanalytics

## Abstract

DNA-Microarrays have become a potent technology for high-throughput analysis of genetic regulation. However, the wide dynamic range of signal intensities of fluorophore-based microarrays exceeds the dynamic range of a single array scan by far, thus limiting the key benefit of microarray technology: parallelization. The implementation of multi-scan techniques represents a promising approach to overcome these limitations. These techniques are, in turn, limited by the fluorophores’ susceptibility to photobleaching when exposed to the scanner’s laser light. In this paper the photobleaching characteristics of cyanine-3 and cyanine-5 as part of solid state DNA microarrays are studied. The effects of initial fluorophore intensity as well as laser scanner dependent variables such as the photomultiplier tube’s voltage on bleaching and imaging are investigated. The resulting data is used to develop a model capable of simulating the expected degree of signal intensity reduction caused by photobleaching for each fluorophore individually, allowing for the removal of photobleaching-induced, systematic bias in multi-scan procedures. Single-scan applications also benefit as they rely on pre-scans to determine the optimal scanner settings. These findings constitute a step towards standardization of microarray experiments and analysis and may help to increase the lab-to-lab comparability of microarray experiment results.

## 1. Introduction

DNA microarrays have become a powerful tool for systematic monitoring of gene regulation. The technology is based on the competitive hybridization of differentially fluorophore-labeled cDNA-probes with spotted, immobilized DNA-targets. The cDNA’s are transcribed from mRNA acquired from different regulatory states of the chosen biological sample. Thus, the ratio of the immobilized fluorophores on a spot reflects the relative abundance of RNA of the regulatory states under study. Within the last two decades the aforementioned principle has gained widespread use in fields such as molecular biology, genetics, and medicine [1,2]. It allows for the high-throughput transcriptome analysis of transcriptome regulation from a few dozens of genes up to the whole genome of the organism of interest [3].

The vast possibilities this technology provides are evenly met by technical, biochemical, and statistical difficulties. Each step of a microarray experiment introduces new factors that influence and possibly bias the final data. Beginning with choice of sample recovery and primer design, which might cause sequence-dependent bias [4]. Furthermore, the used spotting technique, as well as the choice of buffer, spotting, incubation and washing conditions, all influence spot geometry and uniformity by affecting drop dying and hybridization efficiency [5,6,7,8,9]. Data acquisition is facilitated using laser scanners controlled by PC software. Here, influencing factors are the scanner and it’s lasers themselves [10,11,12], the choice of fluorescent dye [13] as well as the scan settings, especially the scan power and the photomultiplier tube’s (PMT) voltage [14,15], and also exposure to environmental light, ozone, and laser light prior to the data acquisition [11,16,17]. While this multitude of factors does not hinder the acquisition of significant data, it is a major barrier for lab-to-lab reproducibility, comparability, and consistency of microarray experiment data [18].

In order to overcome these limitations a vast array of tools has been developed. Some factors are addressed by changing the experimental design, e.g., additionally using reverse dye assignments (*dye swap*) to account for dye bias [19]. Several techniques focus on the data acquisition itself. Finding the optimal scanner settings has been the subject of a lively discussion [14]. Regardless of the respective settings, all single scan approaches suffer from a limited dynamic range of measured intensity, as the dynamic range of fluorescence intensity exceeds the dynamic range of a single array scan by far [14]. Two basic approaches have been suggested to overcome these limitations. Mathematical or statistical approaches try to correct for saturation or noise using information inherent in the acquired data. Gupta *et al.* [20] for example devised a Bayesian hierarchical model that corrects signal saturation based on pixel intensities. Most approaches however extend the scanning routine by recording multiple scans with different settings. The benefits of multiscan techniques for extending the linear signal range were, among others [21], shown by Khondoker *et al.* [10] who are using a maximum-likelihood-estimations model based on a Cauchy distribution to account for saturated signals and systematic bias. Ambroise *et al.* [12] characterized a PMT independent optical scanner bias that takes account for scanner specific bias. Based on this, a two-way ANOVA model was devised that accounts for scanner bias as well as saturation and noise through utilization of multi-scan data. Multiscan techniques were shown to increase overall data quality as well as reproducibility in comparison with single scans [15,22]. They can also be used to normalize dye specific bias as an alternative to limited methods based on LOESS/LOWESS and others [14,21,23].

An ubiquitous difficulty when working with fluorophores is photobleaching, an irreversible photochemical reaction which destructs the fluorophores ability to emit photons [24]. Photobleaching is caused by photons and ozone and differs from fluorophore to fluorophore [11,16,25]. Satterfield *et al.* [11] showed that microarray scans also bleach the fluorophores when they monitored intensity-changes of cyanine-3 (Cy3) and cyanine-5 (Cy5) serial dilution slides under heavy use over the course of five weeks. These findings imply a possible effect of photobleaching on multiscan data quality.

In this study, we evaluate the photobleaching characteristics of Cy3 and Cy5 as part of solid state DNA microarrays. The effects of initial foreground intensity on the degree of bleaching as well as the effect of laser scanner dependent variables such as the PMT voltage on the imaging are investigated. Several microarray slides with identical layout were manufactured with conditions optimized in a previous study and repeatedly scanned with individual static PMT voltages. Identical 5'-cyanine functionalized single strand DNA was immobilized onto the slides in order to reduce sources of bias, such as the sequence differences or dye-incorporation and hybridization efficiency. The resulting data is used to develop a mathematical model capable of predicting the expected degree of signal intensity reduction caused by photobleaching for each fluorophore individually, depending on the initial foreground intensity, the number of previous scans and the desired PMT voltage in order to allow for the removal of photobleaching-induced, systematic bias in multi-scan procedures.

## 2. Model

Microarray scan imaging is dominated by two processes. Firstly, the immobilized, dye functionalized oligos are irradiated by a laser beam, which induces the emission of lower energy photons from the dyes. As the applied scan power is not varied in this study no closer look is taken at the relation between applied power and dye-emitted photons. However, considering photobleaching, this process is of upmost interest, as the cyanine dye loss of photo activity is photon-induced. Although the mechanism is not completely understood yet, it can be assumed that bleaching affects each cyanine molecule independently. Also, not every excited molecule is bleached. This leads to the assumption that photobleaching can be described as a degradation process, analogue to radioactive decay:

(1) $$\text{p}\left({\text{p}}_{0},{\text{n}}_{\text{scan}}\right)={\text{ p}}_{0}\times {\text{e}}^{-\text{λ }\times \;\left({\text{n}}_{\text{scan}}-1\right)}$$

where *p(p_0,_ n_scan_)*: photons emitted after n scans; *p*_0_: initial photons emitted (*n_scan_* = 1); *n_scan_*: number of scans; λ: degradation constant (neglecting a change of scan power, λ is assumed to be dye specific).

The photons, emitted from the cyanine dyes, are not directly measured by an optoelectronic transducer. They pass the PMT, which acts as a signal enhancer and transducer. In this vacuum tube, the photons strike a photocathode and, as a consequence of the photoelectric effect, electrons are ejected. These electrons again strike a dynode that acts as a multiplier, emitting more secondary electrons. Several dynodes work as a cascade, each holding a higher positive potential than its predecessor and each multiplying its predecessor’s electron signal. Finally, the secondary electrons strike the anode, where the signal is transduced. The extent of signal amplification depends on the voltage setting of the PMT. As multiscan techniques are designed to enlarge the linear signal range of microarray experiments through variation of PMT voltages, it is crucial to characterize and model the PMT voltage’s influence to fully understand its effect on imaging of photobleaching. As a consequence of the previously described cascade effect, the PMT signal enhancement is modeled by an exponential function, similar to Khondoker *et al.* [10]:

(2) $${\text{I}}_{\text{e}}\left({\text{p}}_{0},{\text{n}}_{\text{scan}}\right)=\;e^{\;\times {\text{ p}}_{0}\;\times {\text{ e}}^{-\text{λ }\times \;({\text{n}}_{\text{scan}}-1)}}$$

where *I_e_(p*_0_*, n_scan_)*: post PMT intensity (electron signal); *p*_0_: pre PMT intensity (photon signal, theoretical, not measured).

The above model involves a significant problem: *p*_0_, the emitted photons of the first scan cannot be measured directly. The closest to *p*_0_ is *I*_0_, the post PMT electron signal of the first scan. As described above, the electron signal is an exponential transformation of the photon signal. The exponential relationship cannot be exactly determined. However, transforming the relationship into a linear one by using the natural logarithm of *I*_0_ instead constitutes a practical solution. A model calculated with *ln*(*I*_0_) is valid as long as one stays in the *ln*(*I*_0_)-based reference system:

(3) $$\text{ln}\left(\text{I}\left({\text{I}}_{0},{\text{n}}_{\text{scan}}\right)\right)=\text{ ln}\left({\text{I}}_{0}\right)\;\times {\text{ e}}^{\left(-\lambda \;\times \;\left({\text{n}}_{\text{scan}}-1\right)\right)}$$

where *I*(*I_0_, n_scan_)*: post PMT foreground intensity after n scans, with given *I*_0_; *I*_0_: initial post PMT foreground intensity (*n_scan_* = 1); *n_scan_*: number of scans; λ: degradation coefficient.

At this time, the model does not directly feature the applied PMT voltage. It might not have to directly incorporate the voltage at all if it’s influence is already sufficiently covered by *I*_0_, which itself is directly dependent on the applied PMT voltage. In case that our model does not account for all major variance in the data an additional parameter is introduced. This parameter must be consistent with our degradation or decay model, e.g., the model should return *I*(*n_scan_*) = *I*_0_ for *n_scan_* = 1. This condition rules out intercepts and coefficients on the linear level of our model. The exponential term cannot be extended by adding an intercept for the same reason. The addition of an exponential coefficient would be redundant as one already exists (λ). Adding an exponent to (*n_scan_* − 1), however, allows for the alteration of the degradation behavior without thwarting the conditions of a degradation model.

The combination of models (1), (2) and (3) together with the abovementioned considerations lead to the following function, which is theoretically suited to model the effect of photobleaching on measured intensities of microarray scans, taking into account the initial measured intensity (*I*_0_), the number of previously executed scans (*n_scan_*):

(4) $$\text{ln}\left(\text{I}\left({\text{I}}_{0},{\text{n}}_{\text{scan}}\right)\right)=\text{ ln}\left({\text{I}}_{0}\right)\;\times {\text{ e}}^{\left(-\text{λ }\times \;{\left({\text{n}}_{\text{scan}}-1\right)}^{\text{a}}\right)}$$

where *I*(*I_0_, n_scan_*): post PMT foreground intensity after n scans, with given *I*_0_; *I_0_*: initial post PMT foreground intensity (*n_scan_* = 1); *n_scan_*: number of scans; λ: degradation coefficient; *a*: exponent.

## 3. Experimental Section

### 3.1. Oligo Preparation

Single strand DNAs (ssDNA) of 40 nt length were purchased from Eurofins Genomics GmbH (Ebersberg, GERMANY). The internally-compiled sequence was optimized with regard to low stabilities of potential homodimers and hairpins. The 5'-end of the ssDNA was modified with a Cy3 or Cy5 respectively. The 3'-end of the ssDNA was modified with an amino-modified C7 spacer: 5' Cy3/Cy5–C ACG ATT CGG CTT TAG GTC AAC TGG ATT TCG GCT TAG GAC–C7-Amino 3'. In order to minimize variance it was decided to use only one sequence, with one spacer-type and a set dye abundance per oligo. Instead of a real hybridization, both Cy5 and Cy3 dyes on nt-identical but mixed DNA pools are printed together as sequence-identical ss-DNA 40-nt strands. While this does not reflect the realities of an actual microarray DNA hybridization experiment, it is suitable to demonstrate the effect of photobleaching as well as it can be used as the basis for quantification. Each oligo was serially diluted with a buffer containing 3× standard saline citrate (SSC) and 0.001% 3-[(3-Cholamidopropyl)dimethylammonio]-1-propanesulfonate (CHAPS) to concentrations ranging from 5 to 0.05 µM (a detailed table can be found in the supplementary materials). The buffer composition was chosen as a result of preliminary tests based on the works of Dawson *et al.* [6] in order to allow for homogenous distribution of the spotted oligos and minimized drying effects, thus minimizing spot heterogeneity (spot homogeneity information can be found in the supplementary materials). Solutions were stored at 4 °C and protected from light.

### 3.2. DNA Immobilization

DNA sequences were immobilized on the aldehyde glass slides (SuperAldehyde 2; Arrayit^®^ Corporation, Sunnyvale, CA, USA) using a non-contact-spotter (Nano Plotter™ NP2.1; GeSiM mbH, Großerkmannsdorf, GERMANY) with an applied voltage of 75 V. The selection of a contactless printer allowed for higher homogeneity in spot geometry by avoiding pin-derived variance. Concentrations between 0 and 5 µM per dye were spotted in various pre-mixed combinations (a detailed table can be found in the supplementary materials). The spotting layout consisted of 2 × 8 blocks, where each block held 1 spot per oligo mixture giving a total of 16 spatially distributed spots per oligo mixture per slide. After drying the slides overnight in the dark, six washing steps using 4× SSPE buffer and water were performed, according to Dawson *et al.* [6].

### 3.3. Data Acquisition

All scans were performed using the GenePix^®^ 4000B Microarray Scanner by Molecular Devices (Sunnyvale, CA, USA). All data was collected at a pixel size of 10 µm and a total resolution of 1891 × 2089 pixels. Spot sizes were 229.48 µm ± 18.77 µm. Model data was acquired subsequently through one preliminary scan to determine the scan area and 20 additional scans per slide with constant PMT settings at 100% scan power, leaving approx. 6 min between the start of two scans. In this first modeling approach it was decided to only use 100% laser power in order to maximize the observable effect. Each slide was scanned with a different PMT setting, displayed in Table 1. Data collection was carried out by using GenePix^®^Pro 6.0 (Molecular Devices, Sunnyvale, CA, USA).

**Table 1:** PMT settings of different DNA chips.

# Chip	PMT_635 nm_ [V]	PMT_532 nm_ [V]
1	950	700
2	850	600
3	750	500
4	650	400
5	550	300

Validation data was acquired subsequently through one preliminary scan to determine the scan area and five additional scans with varying PMT voltage settings at 100% scan power (see Table 2). This independent data set consisted of three chips that were, except for the scanning process, identical in layout and processing to the five model chips.

**Table 2:** PMT settings of validation data.

# Scan	PMT_635 nm_ [V]	PMT_532 nm_ [V]
1	550	300
2	650	400
3	750	500
4	850	600
5	950	700

### 3.4. Data Analysis

#### 3.4.1. Post Processing

In addition to the criteria applied by GenePix^®^Pro in order to flag and exclude low quality spots, all spots with any saturated pixels as well as spot whose signal to noise ratio (SNR) was 3 or lower were excluded from further analysis. The SNR is defined as follows:

(5) $$SNR=\;\frac{m_{Foreground}-m_{Background}}{s_{Background}}$$

where *m*: median; *s*: standard deviation.

Furthermore, following Lyng *et al.*’s recommendations [15], all sets of spots with median foreground intensities of the first scan (*I*_0_) above 50,000 and below 1000 relative intensity units were excluded from further analysis to prevent saturation and/or noise bias. Although a correction for background is a general convention, the actual application varies. Background correction is carried out locally, within a sub-grid, with blank spots or control spots. Most of these approaches have different underlying assumptions on how the background intensity reflects an intensity bias over- or better underlying the feature intensity. Furthermore Qin *et al.* [26] showed that while a background subtraction actually reduces the bias it increases data variability. Furthermore we have to investigate if and how the background intensity changes with increasing scans. If the background is indeed affected the question if the process occurs comparably on the surface of the actual spot still remains. These aspects were the basis of our decision to omit a background correction and to postpone a thorough examination of background photobleaching to future studies. Data conversion and filtering was carried out using the open source program R Studio Desktop v0.99.441 (R Studio, Boston, MA, USA).

#### 3.4.2. Modeling

The processed data was modeled using internally-written scripts in MATLAB v7.12.0.635 (The MathWorks, Inc., Natick, MA, USA).

This model concentrates on actual detected intensity and not on spotted concentration. This decision was made regarding intensity profile heterogeneity of replicate spots of the same concentration (e.g., for Cy5 in this experiment, the average percent intensity deviation for replicate spots was approx. 28.58% ± 20.17%, more information can be found in the supplemental materials). This is a valid approach as the photobleaching depends on the actual amount of bound fluorophore on the spot and working with the intensity instead of the applied concentration allows for modeling without spot intensity profile bias.

At first, a regression was calculated for each independent spot, using the model described in Section 2 for both the Cy5 and Cy3 channel. For these regressions MATLAB’s own non-linear least-square fitting algorithms based on trust regions was applied. Using Cy5 model data with *R^2^* ≥ 0.95 the dependency of both calculated parameters, λ and *a*, on PMT voltage and/or initial intensity was examined. Each variable, voltage and intensity, was examined independently for each parameter (λ and a) by carrying out an analysis of variance (ANOVA). This approach was chosen to determine if a dependency can be observed that introduces a variance into the data, significantly higher (α ≤ 0.01) than the experimental variance for the parameters (“Lack of Fit Test”). To allow for ANOVA analysis of I_0_ dependency, I_0_ data was organized in groups spanning 100 relative intensity units. Each significant dependency was then modeled using second order polynomials.

The acquired Cy5 model parameters were used to calculate a surface fit with the processed Cy3 model data. Cy3 parameters were modeled analogous to their Cy5 counterparts.

#### 3.4.3. Validation

The generated models for both Cy5 and Cy3 photobleaching were applied onto the validation data, which was also processed as described in Chapter 3.3.1. The model term was converted to allow for the calculation of the initial intensity, given the current intensity (*I*(*n_scan_*)), the used PMT voltage, and the amount of scans carried out before. The mean *R^2^* of the linear fits of intensity *vs.* PMT voltage, as well as the standard deviations of the two linear parameters for all uncorrected data series were compared to the same criteria of all corrected data series for each cyanine dye independently.

## 4. Results and Discussion

### 4.1. Regression Analysis

**Figure 1:**
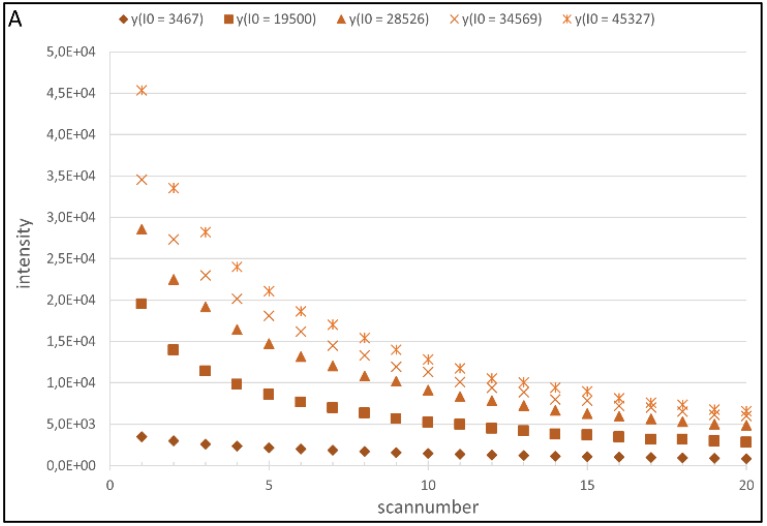

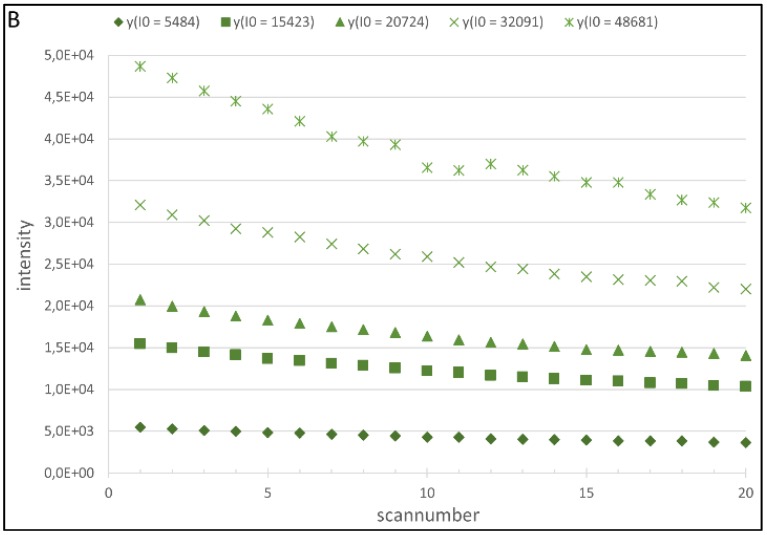
(**A**) Change in measured intensity of Cy5-labeled cDNA spots with increasing number of scans, depending on their initial intensity; and (**B**) change in measured intensity of Cy3-labeled cDNA spots with increasing number of scans, depending on their initial intensity.

Using model (4) with the preprocessed model data (exemplary shown in Figure 1) resulted in different outcomes for the two color channels. While for Cy3 59.9% of 1331 regressions had an *R^2^* of 0.9 and above, 96.5% of all 1772 calculated regressions for Cy5 showed *R^2^* of 0.9 and higher. This discrepancy could be a consequence of the well-known higher background of the Cy3 channel. The model data, however, contradicts this assumption as standard deviations for both channels are of comparable order and the SNR of the Cy3 channel is even higher (132.49) compared to the Cy5 SNR (51.11). Although Staal *et al.* [25] quantified the crosstalk of Cy5 to Cy3 as little as 0.2%, it is still possible, especially at higher PMT settings, that Cy5 crosstalk biases the Cy3 data. As the recorded spots were made of mixtures with varying concentrations of each dye, a spot with a high Cy5 concentration and a low concentration of Cy3 is likely to be biased in a more severely manner. An effect biasing the data could be Förster Resonance Energy Transfer (FRET) between Cy3 and Cy5 and intra spot heterogeneity. The transfer of energy between a donor and an acceptor in close proximity has been well described for nucleotide-bound fluorophores in general, and Cy3 and Cy5 specifically, [27,28]. Through FRET some of the excited cyanines could have transferred the energy to their cyanine counterpart instead of emitting photons, thereby reducing the detected intensity of the respective channel. As FRET is highly dependent on a close proximity of donor and acceptor, this effect will be much more prevalent in high concentration spots or areas of higher nucleotide density in heterogeneous spots. The interdependency of FRET, intra‑spot heterogeneity and photobleaching has been investigated by Rao *et al.* [29,30]. Radial and vertical intra-spot heterogeneity of printed targets profoundly influence local hybridization efficiency and finally the fluorescence signal as well as the occurrence of FRET. The described conjunction could also affect photobleaching rates as the excitation of one cyanine also partially excites the other one, thereby intertwining the exposition to potential photodestruction. Again the possible effect grows depending on the donor and acceptor concentrations. Furthermore Rao *et al.* [29] showed that the destruction of the FRET acceptor (here Cy5) leads to increased emission from the former donor (here Cy3), another source of signal crossover. The process of target-probe hybridization is the major influence modulating the scale of the phenomenon described before. This study’s experimental setup relies on ssDNA printing of directly labeled nucleotides and no hybridization. While FRET and intra-spot heterogeneity can be expected to affect this data as well, the effect of hybridization cannot be accounted for and was subsequently not modeled. Although choice of experimental design regarding FRET complicates the generation of the Cy3 model, it shows that the usage of Cy3 and Cy5, although omnipresent in fluorophore-based bioanalytics, entails limitations that have not yet been properly addressed.

### 4.2. Generation of the Cy5 and Cy3 Model

**Figure 2:**
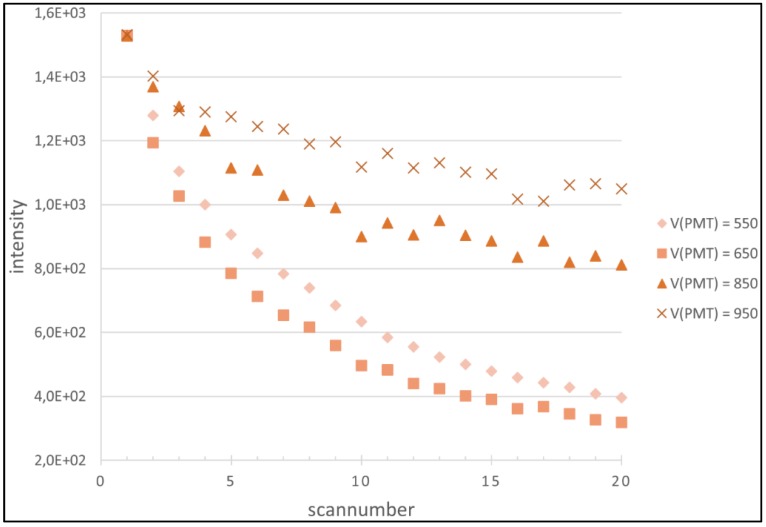
Change in measured intensity of Cy5-labeled cDNA spots of equal initial intensity with increasing number of scans, depending on the PMT voltage.

With respect to these results, it was decided to focus on the Cy5 data for closer examination and to base a more refined model on this data. 96.5% of all 1772 calculated regressions for Cy5 showed *R^2^* of 0.9 and higher and were used to generate the model. A model adjusted for Cy3 is calculated based on the Cy5 model. In order to investigate possible influences of the initial foreground intensity (*I*_0_) and/or the PMT voltage (*V_PMT_*) on both the degradation coefficient λ and the exponent a, multiple analyses of variance (ANOVA) were carried out. The underlying idea is to determine if the variance introduced to the parameters by the variables is significantly distinguishable from the experimental variance. This is a practical approach that does not ask if the variables actually influence our parameters, but if the modeling of any hypothetical influence can significantly improve the accuracy of the model, given the inherent experimental variance of the parameters. Firstly, the influence of I_0_ was investigated: Regarding λ, the null hypothesis (*h*_0_: σ^2^_model_ = σ^2^_experiment_) cannot be rejected for any reasonable significance level α (α_h0 rejected, min_ = 0.9477). For a, the lowest significance level that allows for rejection of h_0_ is even higher (α_h0 = rejected, min_ = 0.9999). As a result, both parameters are not modeled with regard of *I*_0_. For *V_PMT_*, however, results were different: *h*_0_ for λ as well as for a are rejected at an α well below all levels established in applied statistics (λ: α_h0 = not rejected, max_ = 9.09 × 10^−123^, a: α_h0 = not rejected, max_ = 4.08 × 10^−112^). It is contradictory that *V_PMT_*, a variable of a process succeeding the actual bleaching, is supposed to influence the parameter characterizing it. We assume that the PMT voltage’s influence on λ does obviously not display its influence on bleaching itself. A *V_PMT_*-dependent λ is an expression of the transformation of the “observed” bleaching through the imaging process, which itself is *V_PMT_*-dependent. These findings indicate that the variance introduced to the model data through *V_PMT_* cannot be completely modeled indirectly using I_0_ alone, which is directly V_PMT_-dependent. The effect of *V_PMT_* is clearly visible in the model data (see Figure 2).

All in all, modelling of both parameters including V_PMT_ might yield a significant benefit in accuracy and it is therefore carried out and applied to our Cy5 model:

(6) $$ln\left(I\left(I_{0},n_{scan},\;V_{PMT}\right)\right)=\;ln\left(I_{0}\right)\;\times e^{\left(-\lambda \left(V_{PMT}\right)\;\times \;{\left(n_{scan}-1\right)}^{a\left(V_{PMT}\right)}\right)}$$

where *I*(*I_0_, n_scan_, V_PMT_*): post PMT foreground intensity after n scans, with given *I*_0_ and *V_PMT_*; *I_0_*: initial post PMT foreground intensity (*n_scan_* = 1); *n_scan_*: number of scans; *V_PMT_*: PMT voltage; *λ*(*V_PMT_*): degradation coefficient; *a*(*V_PMT_*): exponent.

Both λ(*V_PMT_*) and *a*(*V_PMT_*) were modeled using second order polynomials. Based on the Cy5 model, a fit for Cy3 model data was calculated by varying λ and a for each *V_PMT_* setting. The resulting parameters were examined using ANOVAs analogous to the Cy5 procedures, yielding comparable results. The *V_PMT_* influence was then modeled using second order polynomials. The results are given in term (7) and (8) as well as table 3:

(7) $$\lambda \left(V_{PMT}\right)=\;p_{1}\times {V_{PMT}}^{2}+p_{2}\times \;V_{PMT}+p_{3}$$

where λ(*V_PMT_*): degradation coefficient; *V_PMT_*: PMT voltage; *p*_1_, *p*_2_, *p*_3_: paramters.

(8) $$a\left(V_{PMT}\right)=\;p_{1}\times {V_{PMT}}^{2}+p_{2}\times \;V_{PMT}+p_{3}$$

where *a*(*V_PMT_*): degradation exponent; *V_PMT_*: PMT voltage; *p*_1_, *p*_2_, *p*_3_: paramters.

**Table 3:** Parameters of the final fits.

Fluorophore	λ *(V_PMT_)*			*a(V_PMT_)*		
	p_1_	p_2_	p_3_	p_1_	p_2_	p_3_
Cy3	−2.153E−07	3.232E−04	−9.200E−02	1,106E−06	−1.885E−03	1.461
Cy5	−1.122E−08	1.640E−5	−1.948E−03	−4.533E−07	9.433E−05	0.901

### 4.3. Model Analysis

**Figure 3:**
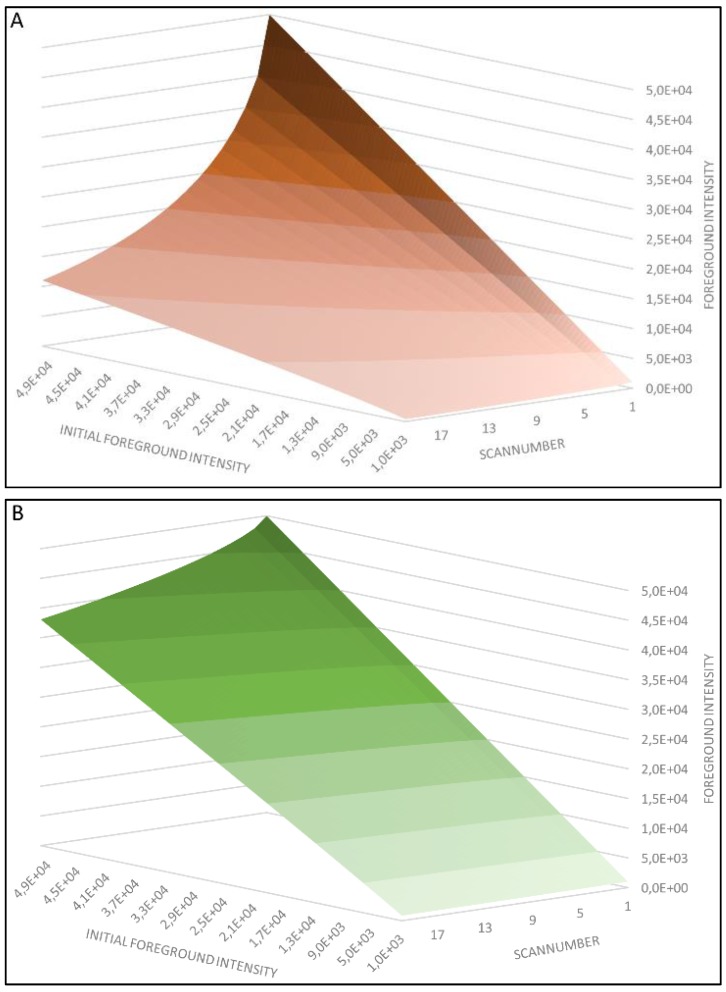
(**A**) Three-dimensional illustration of the final model of Cy5-photobleaching for *V_PMT_* = 950 (**B**) Three-dimensional illustration of the final model of Cy3-photobleaching for *V_PMT_* = 700.

**Figure 4:**
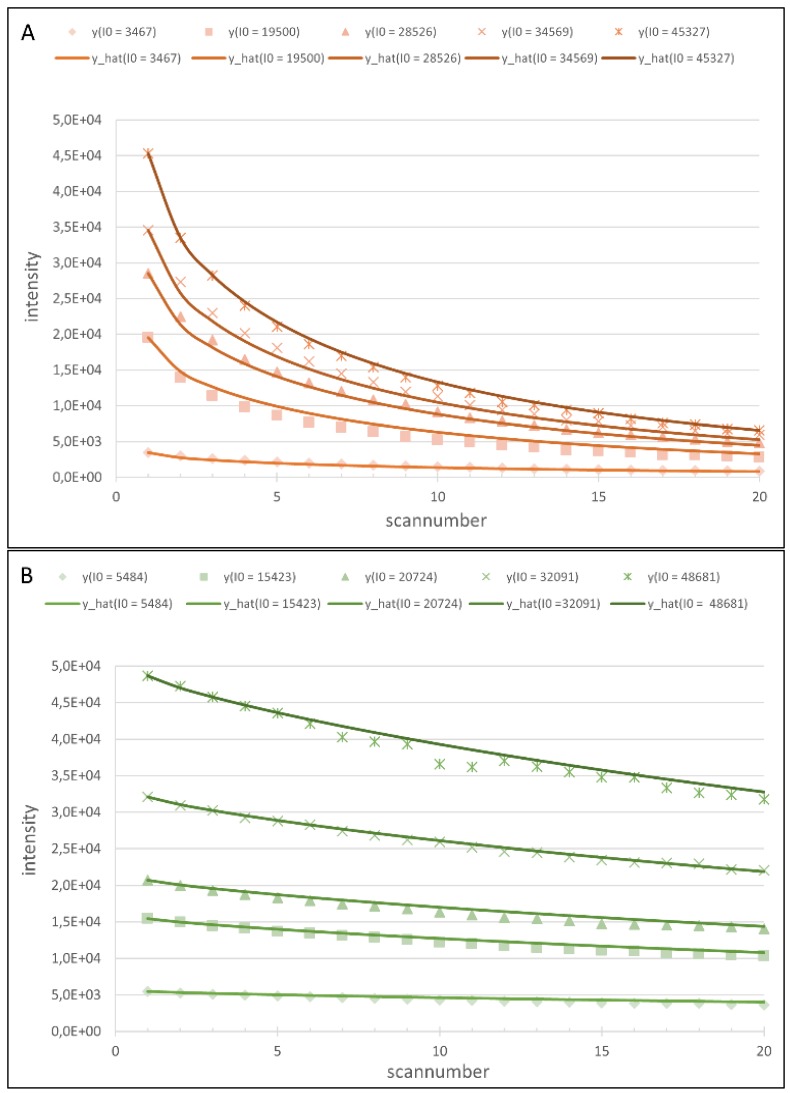
(**A**) Cy5-data sets (*y(I0)*) with model-data (*y_hat(I0)*) at *V_PMT_* = 750. *R^2^*: 0.994, 0.995, 0.998, 0.998, 0.999 (from lowest I0 to highest); and (**B**) Cy5-data sets (*y(I0)*) with fits (*y_hat(I0)*) at *V_PMT_* = 500. *R^2^*: 0.994, 0.997, 0.991, 0.995, 0.984 (from lowest I0 to highest).

Both resulting models (shown in Figure 3) describe the observed bleaching effects to a high degree (*R^2^* from 0.976 to 0.998 for different V_PMT_ settings, examples shown in Figure 4). The unequal susceptibilities of Cy3 and Cy5 to photobleaching clearly stand out: While Cy3-tagged spots lose between 23.19% and 32.01% of their observed intensity after 20 scans, the intensity of Cy5-tagged spots decrease between 76.92% and 87.07%. As can be seen in the model, the variance in signal decrease is introduced by the V_PMT_ settings, which shows that its incorporation into the model is crucial to remedy bias caused by bleaching. Looking at a scan number more likely to be utilized in daily microarray analysis, even after 5 scans the effect profoundly influences the observed intensities: Decreases of 8.73%–10.43% for Cy3 and 41.77%–52.97% for Cy5 emphasize the need for photobleaching correction and scanning protocol standardization not only for multiscan techniques, but for every application relying on microarray scan imaging. Furthermore, the dye-dependent bleaching-variation calls for a re-evaluation of dye swap and dye switch applications as well as mathematical tools designed to compensate for dye introduced bias (LOESS/LOWESS).

### 4.4. First Model Validation

Following the model generation and characterization a model-based correction for photobleaching was carried out. The source data for this procedure (validation data) was recorded in a manner designed to emulate a random multiscan procedure. The slides used were manufactured analogous to their model data counterparts.

A basic principle of multiscan procedures lies in the correction of saturated or noisy spots through extrapolation of intensity data of different V_PMT_ settings. The reliability of the related extrapolation model is based on how well-defined its parameters are. In order to get a first assessment of the effect of photobleaching correction onto parameter quality, linear fits were calculated for data series of the same spots with differing V_PMT_. Fits were calculated for each cyanine dye separately, with raw validation data and model corrected validation data. As seen in Table 4, the application of our model reduces the overall variability (σ_coefficient_, σ_intercept_), thereby improving the data’s suitability for generating an extrapolation model (*R^2^*). The overall low coefficients of determination imply that a reasonable amount of variation remains. While the data was filtered in terms of noise and saturation, other source for variation were not addressed e.g., background intensity. No background correction was applied to the utilized data, as the background itself might be subject to photobleaching. This, and the ongoing discussion if the subtraction of background intensity, is actually beneficial in terms of variability reduction [26] were the reasons for refraining from any background normalization. The characterization of the effect of photobleaching to the background will be the subject of future investigations.

**Table 4:** Comparison of regression features of linear fit of *ln*(*I*) *vs.* V_PMT_ for raw validation data and model corrected validation data for Cy5 and Cy3. Displayed are the mean *R^2^* as well as the mean σ for both parameters of the linear fit for both cyanine dyes for uncorrected and corrected validation data.

Fluorophore	Regression Feature	Data Source	
		Raw Validation Data	Model Corrected Validation Data
Cy5	$\bar{R^{2}}$	0.825	0.8384
	${\bar{\sigma }}_{coefficient}$	44.222	26.429
	${\bar{\sigma }}_{intercept}$	$2.695\;\times 10^{4}$	$1.120\times 10^{4}$
Cy3	$\bar{R^{2}}$	0.818	0.833
	${\bar{\sigma }}_{coefficient}$	81.908	29.613
	${\bar{\sigma }}_{intercept}$	$5.038\times 10^{4}$	$1.258\times 10^{4}$

## 5. Conclusions

Our aim was to characterize and quantify the impact of photobleaching for DNA microarrays. Several groups have previously published approaches to improve the quality and capability of DNA microarray experiments, especially the extension of the linear range through multi-scan protocols constitutes a promising tool. We identified and characterized a major bias for multi-scan procedures and present a way to correct for this bias. In summary, we were able to generate models that explain photobleaching induced variability in multiscan microarray experiments for the two most commonly used fluorophore dyes, Cy3 and Cy5. Our models take into account the initial foreground intensity (*I*_0_), the number of carried out scans (*n_scan_*) as well as the current intensity (*I*) recorded with a defined PMT voltage (*V_PMT_*). Parallel to the generation of these models we characterized the photobleaching effect of both abovementioned dyes, demonstrating the need for correction of this phenomenon not only for multiscan applications, but for all microarray scan based methods, e.g., our model, which explains the variability to a highly significant level and shows that the bleaching, itself, is not a simply linear subtractive effect. We therefore assume that a mere correction of the dye effect does not correct for the photobleaching by which the spots have been affected. A dye swap will in fact correct for intensity differences introduced by the choice of dye, but if the spots also differ in intensity, which they almost always will to a certain degree, photobleaching will not be automatically be co-corrected as it is not a linear additive effect. The degree of influence this effect has on microarray scans, and its disparity depending on the involved dye and the intensity level therefore calls for re-evaluation of dye swap/switch applications and dye effect normalization methods. As photobleaching is, to a lesser degree, induced by environmental light and other environmental factors, such as ozone concentration, our results suggest a standardization of microarray-slide handling to achieve comparable, if possible, minimal exposition to light prior to the scanning process. We are aware that a total lab-to-lab comparability in terms of microarray processing is not realistic, but still want to address the influence of environmental factors on bleaching and the overall quality of microarray results. A real standardization will not be accomplished by one single step, but through raising awareness of the subject we hope to help improve the reproducibility within a lab/workgroup. The benefit of correcting photobleaching-induced variability in multiscan applications was demonstrated. Corrected data was more suitable to generate linear *ln*(*I*) *vs. V_PMT_* fits, leading to more narrowly defined parameters. Future studies need to validate these findings for actual hybridization experiments with dye-functionalized cDNA, accounting for the hybridization-derived effects on photobleaching involving the inclusion of the interdependent factors of intra-spot heterogeneity and FRET and non-FRET crosstalk. Several other factors need to be evaluated to apply our findings to DNA hybridization experiments in general. Among these the influence of temperature, DNA chain sequence and rigidity, dye concentration, and dye stacking. The overall physico-chemical characteristics of surface bound oligonucleotides are still to be sufficiently characterized [8,31]. Also the effect of photobleaching on background intensity needs to be examined to allow for integration of background correction. Likewise, interactions with other normalization methods have to be evaluated.

We encourage users of the technology to apply this information and develop multiscan solutions that correct for photobleaching.

## Supplementary Files

Supplementary File 1
