# Supplementary material for: The Impact of Photobleaching on Microarray Analysis
Source: Biology (Basel). 2015 Sep 11;4(3):556–72. doi: 10.3390/biology4030556 (PMC4588150; doi:10.3390/biology4030556)
Supplement: Supplementary File 1 [file biology-04-00556-s001.pdf]

Supplemental Materials

Table S1. Loading pattern of 96-well plate which mirrors the spotting pattern. Concentrations are given for Cy3 and Cy5 labeled oligonucleotides. Concentrations were given by the manufacturer and checked via Nanodrop.

# ID	Well	c_{Cy5} [µM]	c_{Cy3} [µM]	# ID	Well	c_{Cy5} [µM]	c_{Cy3} [µM]
1	A1	5.00	0.00	27	C5	0.25	0.50
2	A2	0.00	5.00	28	G5	0.13	0.25
3	A3	0.05	5.00	29	C7	0.05	0.10
4	A4	5.00	0.05	30	C8	2.50	1.25
5	A5	0.05	2.50	31	C9	1.00	0.50
6	A6	2.50	0.05	32	G4	0.50	0.25
7	A7	0.25	2.50	33	C11	0.25	0.13
8	A8	0.10	1.00	34	C12	0.10	0.05
9	G7	0.05	0.50	35	D1	0.75	0.00
10	G3	2.50	0.25	36	D2	0.00	0.75
11	A11	1.00	0.10	37	D3	2.50	2.50
12	A12	0.50	0.05	38	D4	1.00	1.00
13	B1	2.50	0.00	39	D5	0.75	0.75
14	B2	0.00	2.50	40	D6	0.50	0.50
15	B3	0.50	2.50	41	G8	0.05	0.25
16	B4	0.20	1.00	42	D8	0.10	0.10
17	B5	0.10	0.50	43	D9	0.05	0.05
18	G6	0.05	0.25	44	E1	0.50	0.00
19	B7	2.50	0.50	45	E2	0.00	0.50
20	B8	1.00	0.20	46	F1	0.25	0.00
21	B9	0.50	0.10	47	F2	0.00	0.25
22	B10	0.25	0.05	48	G1	0.10	0.00
23	C1	1.00	0.00	49	G2	0.00	0.10
24	C2	0.00	1.00	50	H1	0.05	0.00
25	C3	1.25	2.50	51	H2	0.00	0.05
26	C4	0.50	1.00				

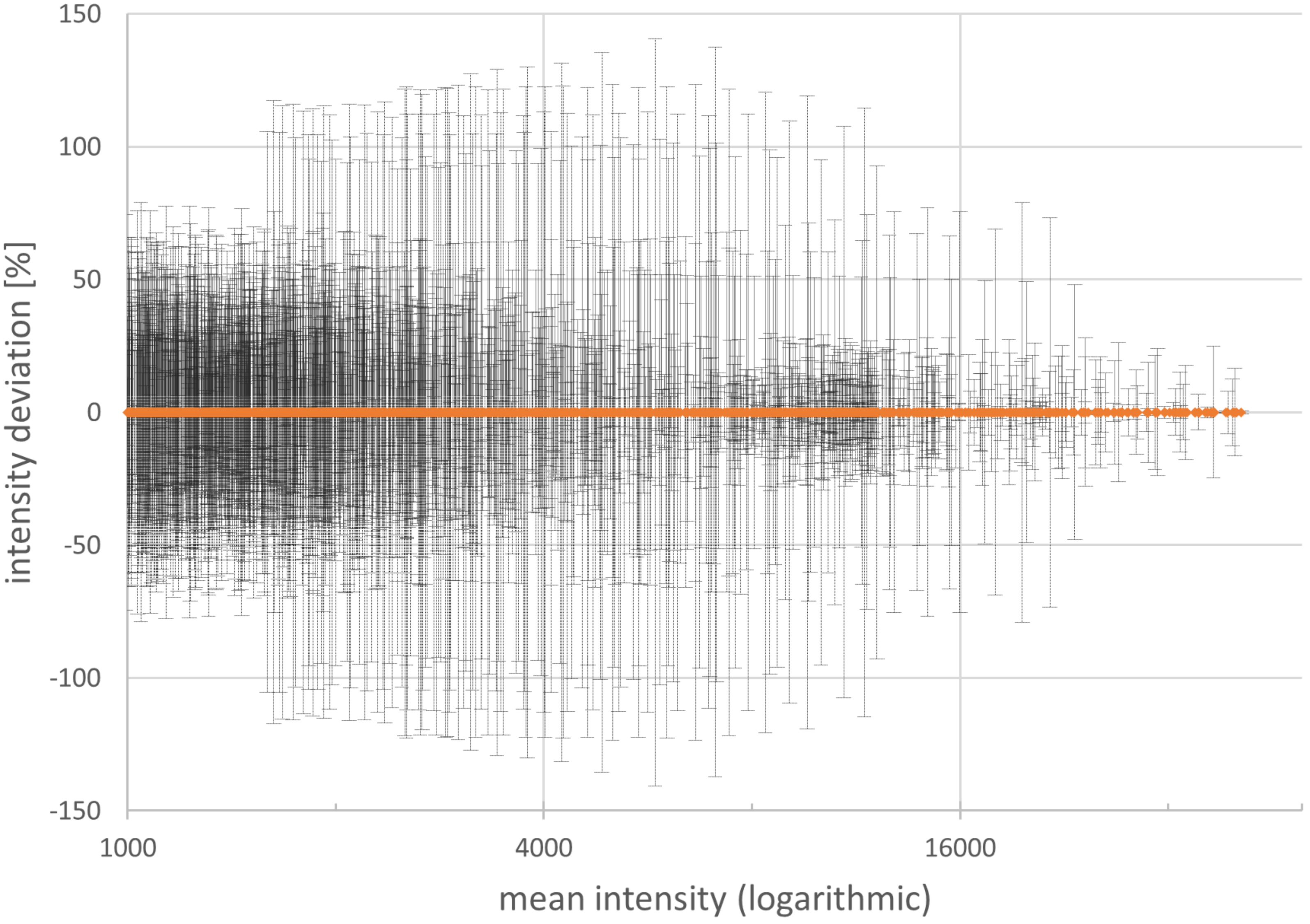

Figure S1. Cy5-intensity pattern heterogeneity of replicate spots. The mean intensities of replicate-spot-groups are displayed with given percent intensity deviation.

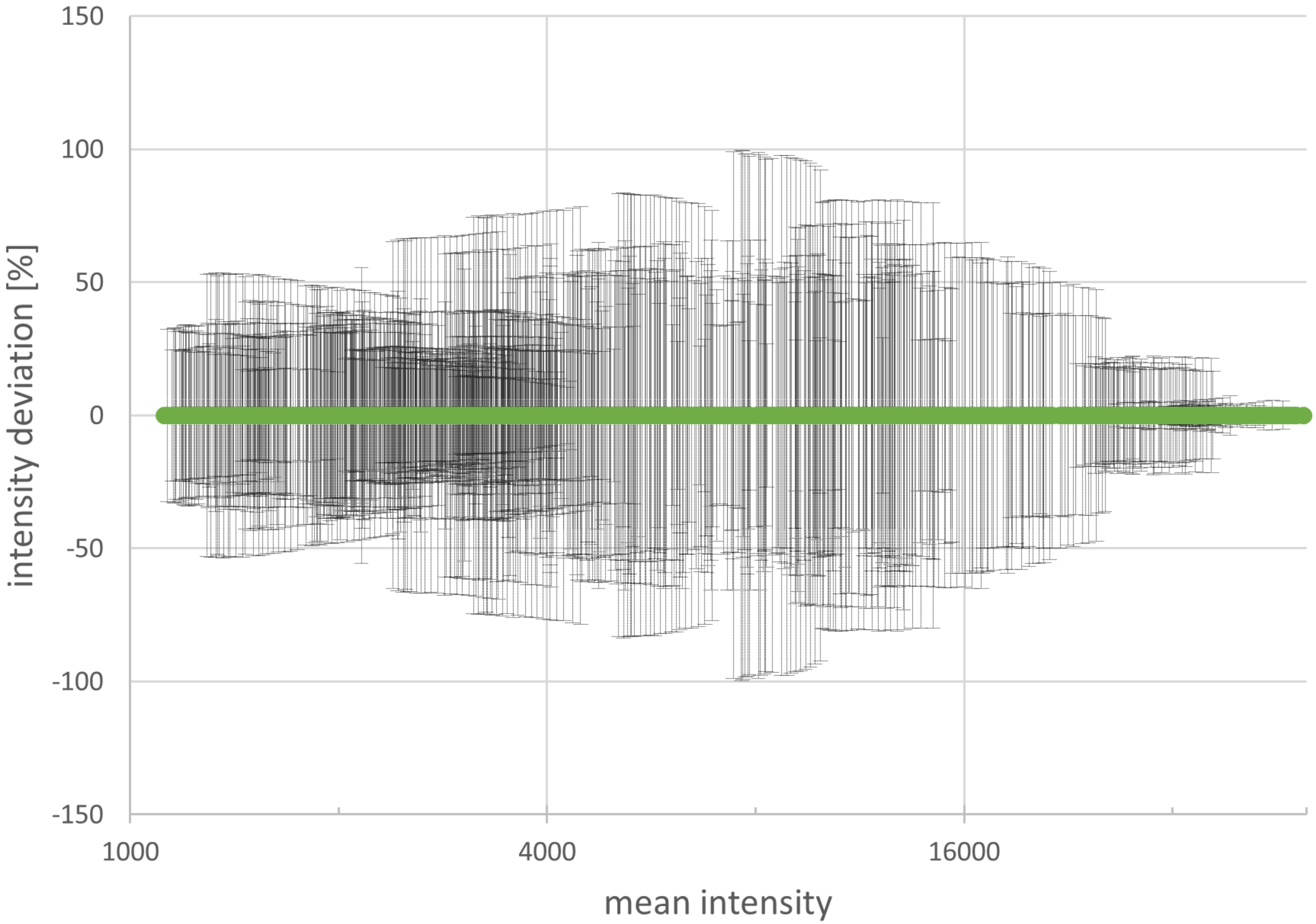

Figure S2. Cy3-intensity pattern heterogeneity of replicate spots. The mean intensities of replicate-spot-groups are displayed with given percent intensity deviation.
